# Developing a framework for understanding policy decision-making behaviors in the transition of a HIV prevention program towards sustainability: a case study from Zambia’s voluntary medical male circumcision program

**DOI:** 10.12688/gatesopenres.15189.1

**Published:** 2024-03-01

**Authors:** Nishan Gantayat, James Baer, Alok Gangaramany, Steve Kretschmer, Rasi Surana, Alick Samona, Njekwa Mukamba, Bright Jere, Tina Chinsenga, Ram Prasad, Stephen Goetschius, Saransh Sharma

**Affiliations:** 1 Final Mile Consulting, New York, New York, 10007, USA; 2 DesireLine, Istanbul, Turkey; 3 Center for Infectious Disease Research in Zambia, Lusaka, Lusaka Province, Zambia; 4 Ministry of Health, Zambia, Lusaka, Zambia

**Keywords:** decision-making, policy-making, organisational behavior, policy implementation, public health, zambia, VMMC, HIV prevention

## Abstract

Faced with declining donor funding for HIV, low- and middle-income countries must identify efficient and cost-effective ways to integrate HIV prevention programs into public health systems for long-term sustainability. In Zambia, donor support to the voluntary medical male circumcision (VMMC) program, which previously funded non-governmental organizations as implementing partners, is increasingly being directed through government structures instead. We developed a framework to understand how the behaviors of individual decision-makers within the government could be barriers to this transition. We interviewed key stakeholders from the national, provincial, and district levels of the Ministry of Health, and from donors and partners funding and implementing Zambia’s VMMC program, exploring the decisions required to attain a sustainable VMMC program and the behavioral dynamics involved at personal and institutional levels. Using pattern identification and theme matching to analyze the content of the responses, we derived three core decision-making phases in the transition to a sustainable VMMC program: 1) developing an alternative funding strategy, 2) developing a policy for early-infant (0-2 months) and early-adolescent (15-17 years) male circumcision, which is crucial to sustainable HIV prevention; and 3) identifying integrated and efficient implementation models. We formulated a framework showing how, in each phase, a range of behavioral dynamics can form barriers that hinder effective decision-making among stakeholders at the same level (e.g., national ministries and donors) or across levels (e.g., national, provincial and district). Our research methodology and the resulting framework offer a systematic approach for in-depth investigations into organizational decision-making in public health programs, as well as development programs beyond VMMC and HIV prevention. It provides the insights necessary to map organizational development and policy-making transition plans to sustainability, by explaining tangible factors such as organizational processes and systems, as well as intangibles such as the behaviors of policymakers and institutional actors.

## Introduction

HIV prevention and treatment efforts in low- and middle-income countries have been funded by national governments, bilateral funding, multilateral assistance, and private philanthropic organizations. But funding trends have changed in recent years. Despite international commitments to invest at least $26.2bn annually in low- and middle-income countries by the end of 2020 ^1^, the resource pool amounted to $21.7bn in 2020, leaving a significant gap in funding ^2^. Donor funding to these countries decreased from a high of $8.6bn in 2014 to $8.2bn in 2020 ^2^.

Among the HIV programs affected by these funding challenges is voluntary medical male circumcision (VMMC), a relatively inexpensive, one-off procedure that reduces by 60% a man’s risk of acquiring HIV via unprotected vaginal intercourse ^3^. The World Health Organization (WHO) and the Joint United Nations Programme on HIV/AIDS (UNAIDS) have recommended VMMC for HIV prevention in 15 sub-Saharan African countries with high HIV prevalence and low rates of male circumcision ^4^.

In the face of plateaued funding from donors, countries most affected by HIV find themselves under pressure from donors to identify alternative, sustainable strategies for funding and implementation. In the case of VMMC – a cost-effective prevention intervention that must otherwise compete for resources with increasingly expensive treatments – this means identifying how to route funding through government finance mechanisms and best integrate VMMC implementation into standard health-service provision, while eliminating or reducing reliance on support from VMMC implementation partners, in order to build ownership and program sustainability. This requires a change from the VMMC demand and service-delivery models used until recently, where VMMC has largely been promoted and delivered through structures parallel to national health systems, using seasonal campaigns led by nongovernmental organization (NGO) implementing partners, directly funded by donors.

As most national VMMC programs have been vertically organized, with implementation supported by donors and NGO implementing partners, the transition to integrated programming within local contexts and health systems will be significant. Government and policymakers must often make decisions about optimal resource allocation in an environment marked by incomplete information and uncertainty, while considering specific socio-cultural and political contexts. Given the uncertainty of future support, designing effective transition approaches to sustainable VMMC programs must be a priority for countries to reach and maintain target coverage levels and reduce the number of new HIV infections. This means maintaining high VMMC coverage while strengthening local governance, management, and execution capabilities in the face of the anticipated contraction in resources. A framework would be useful to guide the process by identifying key barriers to such a transition and mapping the decision-making and collaboration required among stakeholders, including donors, implementing partners, and ministry offices at the national, provincial and district levels.

In order to assist the transition to a sustainable VMMC program in Zambia, one of the 15 priority VMMC countries, we sought to understand and identify the priority decisions that are being taken in a range of contexts to formulate policy for transitioning the program. This transition will ideally be from a program that is, top-down, supply-side focused, and donor-funded and donor-directed to one that is government-owned and -led, provides avenues for bottom-up decision-making in policy development, and where decisions are aligned with the goal of long-term sustainability. The key pillars of decision-making identified as leading to sustainability in the Transition and Sustainability Plan for VMMC published by Zambia’s Ministry of Health are: leadership and advocacy; governance and coordination; service delivery; communication and client mobilization; monitoring and evaluation; supply chain; and health financing/resource mobilization ^5^. For each pillar, we aimed to map the key stakeholders who are or should be involved in the decisions, the decision processes, and the barriers and enablers of the decisions. This analysis formed the basis for the development of a Barrier Framework summarizing the existing barriers to more effective decision-making dynamics that would facilitate transition to sustainable VMMC programming in Zambia.

While we applied this approach to VMMC, it offers lessons for other public health programs in low- and middle-income countries, especially those funded initially by outside donors, as they seek to become locally contextualized, and transitioned to integrated, sustainable structures.

## Program sustainability and decision-making

Definitions of sustainability have varied over time and based on country-specific dimensions such as capacity, governance, and income ( Table 1). Research on health-program sustainability emphasizes a number of core aspects: capacity to maintain service coverage to continue control of a health problem, capacity of a project to continue delivering benefits ^6^, and delivering appropriate levels of benefits for an extended period after major financial, managerial, and technical assistance from an external donor is terminated ^7^. PEPFAR identifies four components of sustainability: political will, HIV services that meet people’s treatment and prevention needs, efficient health systems, and sufficient financial resources ^8^.

**Table 1.** Components of sustainable programs proposed by World Bank, USAID and PEPFAR.

Source	Sustainability Components
World Bank ^4^	• Continued delivery of services and production of benefits • Maintenance of physical infrastructure • Long-term institutional capacity • Political support
USAID ^5^	• Capacity development • Service delivery continuity
PEPFAR ^6^	• Political will (governance, leadership, and accountability) • Adequate services (national health system, service delivery) • Efficient health systems (strategic investments, strategic information) • Sufficient financial resources (Sustainable financing)

Other key aspects of program sustainability are integration ^9, 10^, institutionalization ^11^, and routinization ^12^. The benefits of health programs combining vertical and horizontal approaches have been recognized, where the degree of integration is determined based on the health system, communities, and context ^9, 10^. Potential influencers of institutionalization and routinization include project design and implementation factors, such as the project goal negotiation process, project effectiveness, financing, and training, among others ^13^. Factors within the organizational setting are institutional capacity, integration with existing programs or services, and program leadership.

Funding and capacity building are both essential ingredients for sustainability. Sustainability depends upon adequate, flexible, and reliable funding, as well as political support and programmatic control ^14, 15^. Brazil and Romania are examples of countries that have transitioned from being Global Fund beneficiaries and are now self-sufficient in funding ^16^. South Africa and India have progressively increased their domestic contribution to HIV programs to 90% and 70%, respectively ^14^. South Africa was the first country under the PEPFAR partnership framework to transition to full financial responsibility for its HIV program ^17^. This involved capacitating indigenous organizations, creating shared decision-making structures, and facilitating partnerships with public-health facilities, with the help of PEPFAR-funded staff and infrastructure. In Rwanda, international NGOs helped with administrative and programmatic capacity-building of institutions involved at all levels of the HIV response in preparation for the transition of management of PEPFAR-funded programs ^18^. Between 2009 and 2013, the Bill & Melinda Gates Foundation drove the transitioning of its Avahan HIV/AIDS prevention initiative in India from being heavily donor-funded to being predominantly owned and run by the national government ^19^. In the context of Zambia, antiretroviral services for people living with HIV have been quickly and appreciably scaled up through the strengthening of primary healthcare facilities ^20, 21^. Other sustainability initiatives in the sub-Saharan region include in Ghana, where health-system based factors have contributed to the integration of tuberculosis and HIV services using organic funding ^22^, in Zimbabwe to create a community-based HIV care program ^23^, and an HIV risk reduction behavioral intervention for HIV seropositive and sero-discordant couples in Zambia, using a “train-the-trainer” model ^24^.

When considering the strategic decision-making involved in transitioning an HIV prevention program to sustainability, organizational structure can have a profound effect ^25, 26^. In behavioral theories of firms, viewing decisions as an outcome of well-sequenced behaviors, and linking firm-level models to empirical observations of decision outcomes as well as process structure, marks a deviation from the standard economic theory of firms ^27^. This theory assumes individuals and group structures within organizations have complete information about the options available for choice, perfect foresight of the consequences of their choices, and the wherewithal to optimize and identify an option which maximizes utility ^28, 29^. In addition, the rational actors work within well-structured problems and contexts. By contrast, behavioral theories of firms posit that these assumptions are seldom accurate. Organizations often face ill-structured decision contexts where the issues are complex, difficult to define, and lack clear boundaries, while foresight and the consequences of choices are clouded by uncertainty.

The fields of organizational learning theory ^30– 33^ and evolutionary economics ^34^ show how events and experiences drive decision-making that changes organizational behavior. The processes involved in decision-making can be seen as a kind of assessment, where objectives of relevance, implications, coping potential, and normative significance play a critical role in how individuals evaluate potential behavior or decisions ^35^. In the context of complex decisions, the mechanisms behind decision-making within organizations involve behavioral biases and heuristics. These can be explained by behavioral principles such as bounded rationality and the dominant coalition. Bounded rationality refers to the fact that problem-solving is constrained by the limits of people’s cognitive abilities, that people seldom make choices that are in their long-term interest, and that they do not always pursue self-interest. The dominant coalition means that even though their members have differing interests, organizations seem to move with a sense of shared intentionality, similar to the behavior seen in naturally occurring groups ^36^.

Our research was designed to explore and explain how individuals’ behaviors and non-conscious thought processes play out in organizational decision-making in the context of Zambia’s VMMC program and can act as barriers to a successful transition to sustainable programming. Zambia’s VMMC program has made significant progress toward its scale-up goals. By the end of 2018 it had achieved more than 50% of its target of 1.98 million male circumcisions set in the country’s 2016–2020 National Operational Plan for scaling up VMMC ^37^. While VMMC policy, leadership, infrastructure and human resources have been driven by the national Ministry of Health, implementation of the program has been heavily supported by donors and partners, and the Government of Zambia has recognized the need to move from a predominantly campaign-focused, partner-dependent service delivery model to a government-owned and -managed structure ^38^. In view of the shifting priorities as donors reduce their investments, and the changing HIV landscape, the Government of Zambia’s Transition and Sustainability Plan together with the National Operational plan aims to integrate the VMMC program within the Ministry of Health framework ^5, 38^. Our research was designed in collaboration with Zambia’s Ministry of Health to develop a framework for understanding behaviors in the decision-making processes within VMMC policy, and to help the government understand the organizational dynamics at play, in order to assist transition of the VMMC program from being donor-funded and NGO-implemented to a government-owned and government-managed, sustainable structure.

## Methods

The methods discussed here are those used in the first of two phases of research designed to accomplish the goals outlined:

**Phase One: Immersion research** to map the relevant decisions for the VMMC program and the stakeholders involved in those decisions, and understand the various behavioral dynamics that come into play. This phase of research was designed to inform the second phase of work.

**Phase Two: Decision-simulation behavioral research** to develop and test hypotheses about 1) the most relevant barriers to effective decision-making, 2) which decisions these barriers are relevant to, and 3) which decision stakeholders they are relevant to. This second phase indicated which barriers in the system need to be addressed, and how, to realize an effective transition to a sustainable VMMC program in Zambia. The research also aided in finding potential opportunities for process development and structural modifications to VMMC policy decision-making. The methods and results for this phase of research are reported in a separate article.

**Figure 1.**
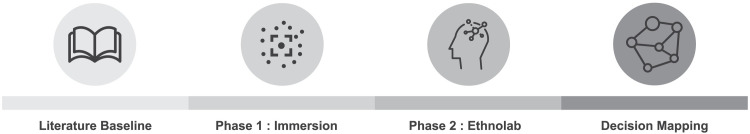
Research process for decision-mapping. Source: Icons – The Noun Project.

The present article focuses on Phase 1: immersion research ( Figure 1).

**Figure 2.**
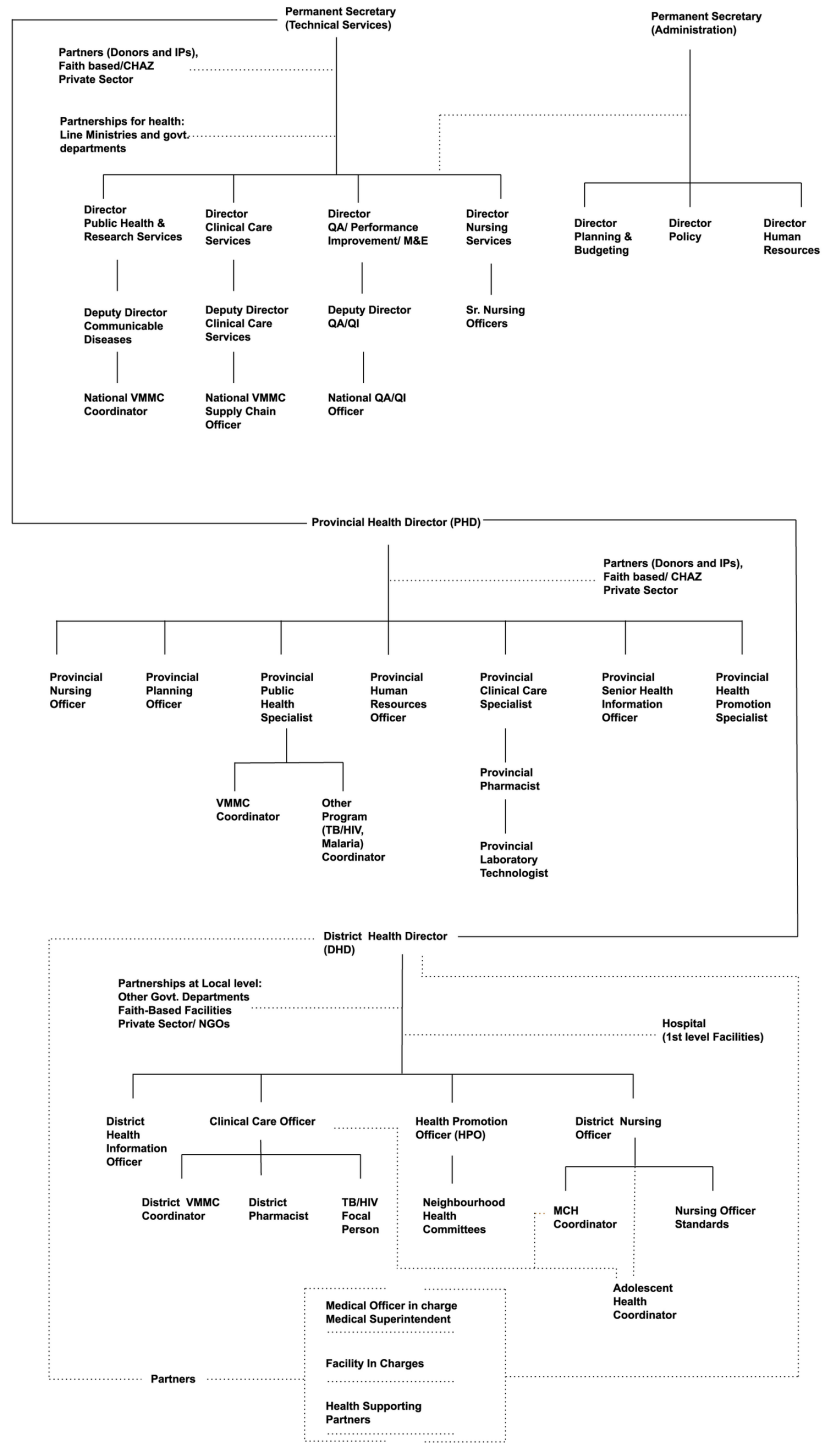
Organogram of Ministry of Health, Zambia. *Note: Provincial and district structures can vary, and the organizational structures are thus indicative of those that pertained during the period of research, January 2020 – October 2020*.

We used a qualitative research technique to identify and understand the mechanisms underlying policy-making decisions and behaviors within the Ministry of Health (MoH) (S2 File, S3 File). We first mapped the organizational structure of the MoH and the roles within it relevant to VMMC program management ( Figure 2), together with other relevant ministries within the Government of Zambia and the relevant roles within them. We then used a purposive sampling technique to select 21 individuals with key roles for the transition of the VMMC program, based on their organizational impact upon health programs within the MoH, and ensuring representation across all three levels of the MoH (national, provincial, and district), as well as implementing partners and donors. We conducted in-person interviews with these 21 individuals: 4 from the national MoH, 8 from provincial MoH offices, 5 from district MoH offices, 2 from implementing partners, and 2 from other ministries. The criticality of their roles with respect to decision-making and engaging across levels was validated during the immersion interviews.

The sample represented two districts in each of three provinces, chosen to represent a range of settings: Mongu and Sioma districts in Western Province (rural setting), Lusaka and Chirundu districts in Lusaka Province (urban setting), and Chinsali and Mafinga districts in Muchinga Province (semi-urban setting). Within each province, one district was selected based on its high VMMC coverage against the 2020 targets, and one was based on its low VMMC coverage. Details of the sample are available in the supporting information (S3 File, Partners+Targets+Dropout Rates tab).

We conducted semi-structured, in-depth interviews to gain a comprehensive understanding of the processes, decision contexts, and organizational structures for policy development and implementation of the VMMC program within the MoH (S1 File). The decision contexts were aligned with the seven “sustainability pillars” in the government’s Transition and Sustainability Plan for VMMC: leadership and advocacy; governance and coordination; service delivery; communication and client mobilization; monitoring and evaluation; supply chain; and health financing/resource mobilization ^5^. Since there was only one respondent for each organizational role, techniques such as focus group interviews were not appropriate, given the lack of within-group heterogeneity. Three researchers – two moderators and one analyst (see extended data) – conducted in-person interviews. Each interview lasted 45–60 minutes and consisted of a set of open-ended, sequenced questions, organized according to a semi-structured discussion guide. The response to each question was recorded using a structured decision sheet (see extended data). All respondents provided verbal consent to be interviewed, and for their interview to be audio-recorded to facilitate data analysis and synthesis. Questions asked during the interview were exclusively about policymaking and decision-making processes. It was explained to respondents that for the purpose of publishing data and results, their responses would be scrubbed of any identifying information. The audio-recorded interviews were transcribed and used for synthesis along with the notes taken during the interview.

**Figure 3.**
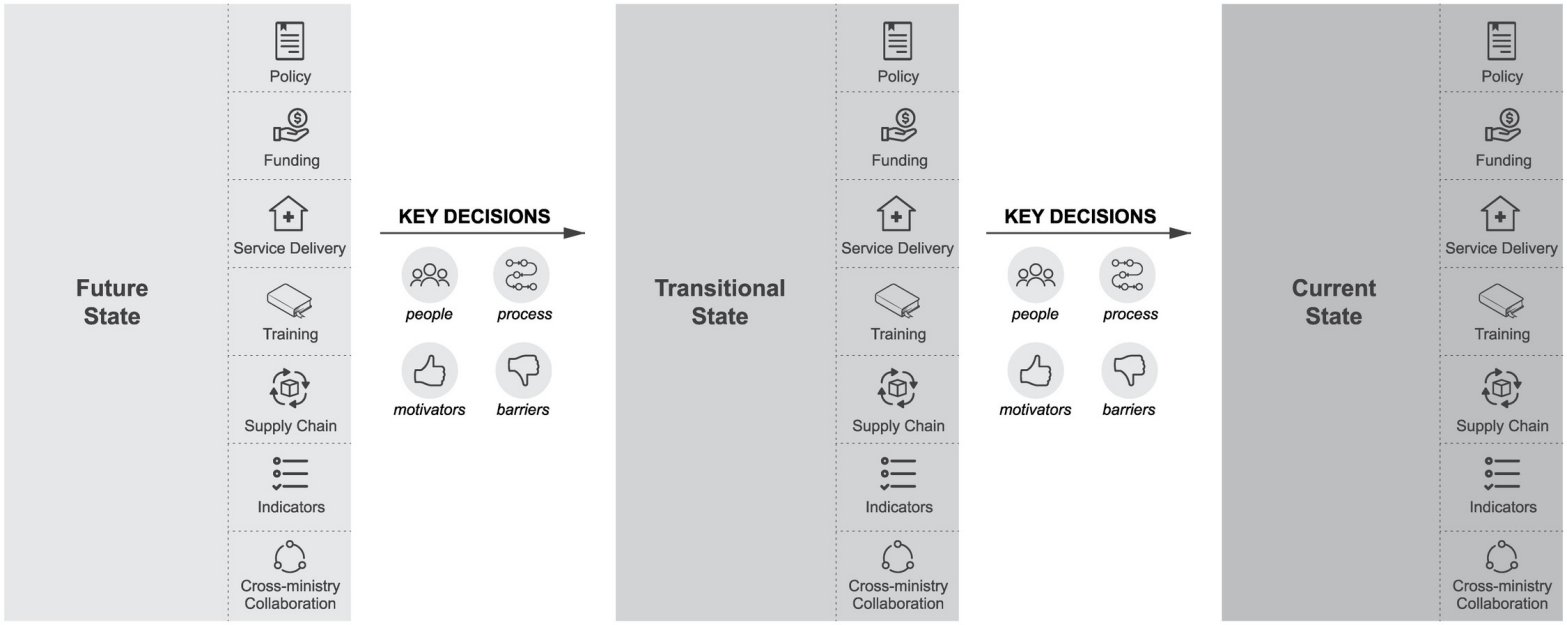
Decision contexts and sequencing of interviews with key decision-makers. Source: Icons – The Noun Project

Interviewers first defined the boundaries of the decision being discussed, by explaining the context and parameters of the decision, and then used a laddering technique to drill down. The participant was asked about the future desired state of policy. For instance, “What is your understanding of sustainability of VMMC?” They were then asked about the decisions that would facilitate the transition to that state. Questions were asked to understand what skills, tools, people, and processes were needed (or were lacking) that would facilitate the decisions leading to this state. Once the future-state and transitional-state decisions were recorded, questions were asked to understand the stakeholders, decision process, and decision flow in more depth, as well as the benchmarks and experiences that might reveal the heuristics that respondents used when making decisions in the current context ( Figure 3). The questions were designed to elicit responses based on participants’ actual experiences and past behavior, rather than their current opinions or feelings (S1 File, S2 File).

One example of a decision context is service delivery. Sample questions about the transition to future desired state were: “ *What might be barriers around service delivery decision-making?*”, “ *What might be some motivations around decisions on service delivery?*”, and “ *What might be the process in decision-making for funding for service delivery?*”. Another such decision context was for decisions taken in the domain of supply chain. The future desired state presented for ensuring a sustainable VMMC policy was a centrally managed supply chain, rather than one managed by donors. Sample questions included: “ *Can you tell me about the decisions around policy that would be needed in the future state of a centrally managed supply chain?*”, “ *What decisions about supply chain might be taking place in the future state where early-infant male circumcision (EIMC) and early-adolescent male circumcision (EAMC) are sustainable and integrated?*”, “ *Who should be involved in making decisions in the future state and what are some of the dynamics involved?*”, and “ *What transitional decisions need to happen before EIMC and EAMC are sustainable?*” Similar questions were then asked about the current system and what might help in the transition from the current structure of the donor-managed supply chain to one managed by the central government. The questions in these examples aimed to elicit answers or narratives based on the respondents’ past experiences in working with service delivery or supply chains within the VMMC program, or in other programs, in order to understand their reference points. For service delivery, for example, partner-led mechanisms might be maintaining the status quo and holding up different and potentially more successful approaches. For supply-chain operations in the national HIV antiretroviral therapy program, the heuristics involved in decision-making might lead to a need for authoritative support when implementing decisions.

To analyze the interviews, we used pattern identification and theme matching to organize the content of the decision sheets (and transcripts of recorded interviews, where available) into a grid. This consisted of seven key decision-making contexts or objectives: VMMC Policy, Funding, Service Delivery, Training, Supply Chain, Indicators, and Cross-Ministry Collaboration. These were broadly aligned with the “sustainability pillars” in the Transition and Sustainability Plan ^5^ and represented key aspects of the overall policy development process. The second dimension of the grid was the structural state of the VMMC program in which a decision occurs: 1) the current, vertically structured VMMC program model; 2) a transitional state, in which decisions are taken and capacities developed to move to a new state; or 3) the future state of a sustainable VMMC program, organically managed by the Government of Zambia, that includes younger age groups (EIMC and EAMC) in order to provide the preventive benefits of circumcision before the young person becomes sexually active. (Since our research was conducted, Zambia’s VMMC program, in line with new recommendations from WHO ^39^, has adjusted the focus of its adolescent programming from boys aged 10 years and older to adolescents aged 15 years and older ^38^.) Each data point from the interviews was mapped to its intersection of the relevant decision-making context and the state. We then synthesized the content for each of these nodes, with a particular focus on the nature of the decision, who is involved, the decision-making process, barriers and enablers to the decision, and the respondent’s point of reference or comparison when describing their decision.

## Results

From the data grid we derived three core decision-making phases (S2 File) in transitioning from the current state to a sustainable VMMC program: (1) alternative funding strategy development, (2) policy development for early infant/adolescent male circumcision, and (3) implementation of VMMC program. Each decision-making phase entails a set of iterative decisions, which are influenced by a range of conditions or behavioral dynamics whose presence or absence facilitates or inhibits decision-making ( Figure 4a, Figure 4b, Figure 4c). Some of these operate in a horizontal dimension (i.e., among ministries at the same level of government); some in a vertical dimension (i.e., among national-, provincial-, and district-level governments or other bodies); and some operate in both dimensions.

**Figure 4a.**
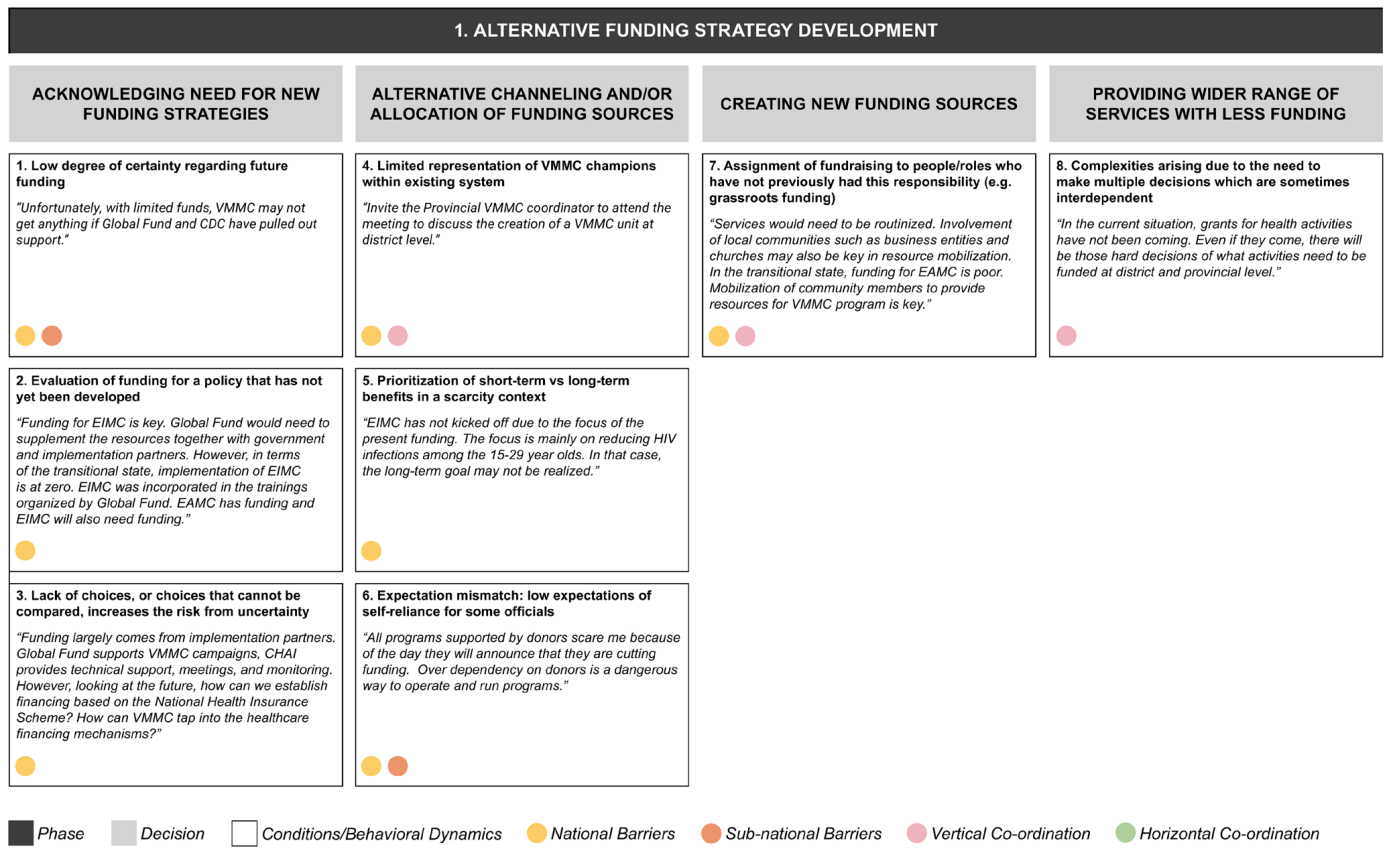
Phase 1 of decision-making and related decision dynamics: alternative funding strategy development.

**Figure 4b.**
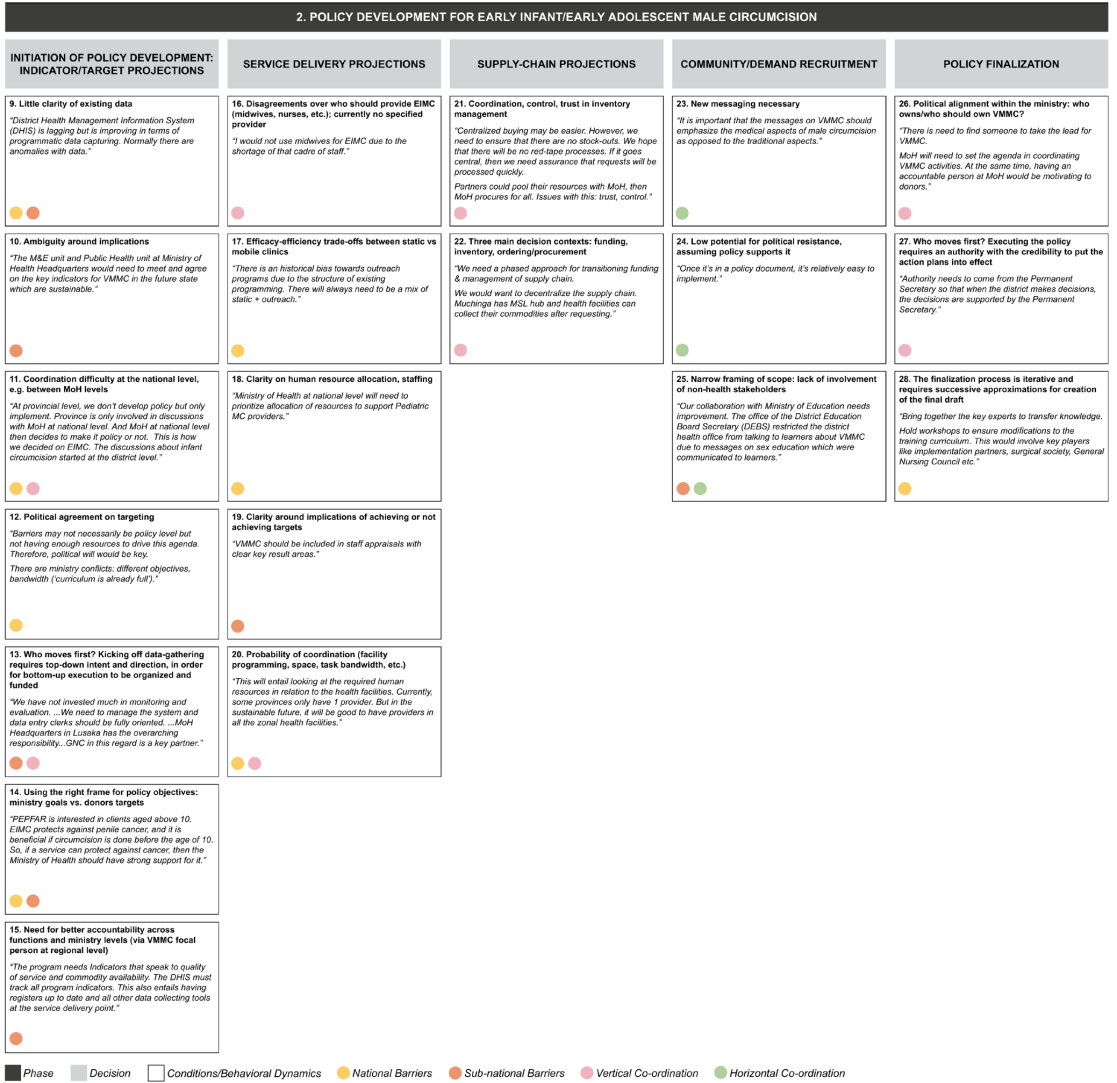
Phase 2 of decision-making and related decision dynamics: policy development for early infant/early adolescent male circumcision.

**Figure 4c.**
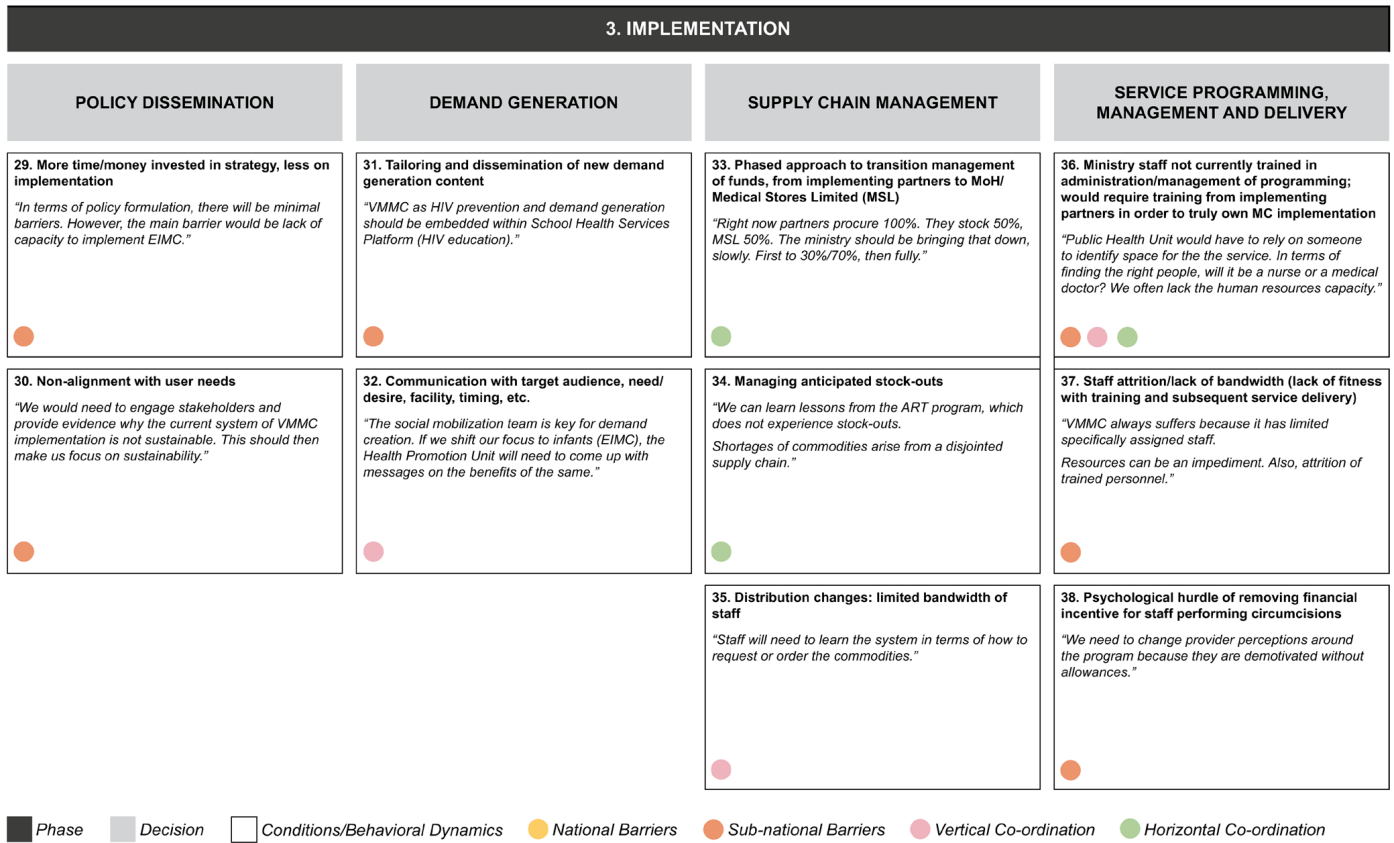
Phase 3 of decision-making and related decision dynamics: implementation.

**1) Alternative funding strategy development** means establishing a reliable funding source and strategy in the absence of external donors. The important decisions for this phase involve acknowledging the need for new funding strategies, allocating funding resources, creating new funding sources, and providing services with reduced funding. Each of these decisions arises in the context of a set of interacting dynamics. Acknowledging the need for new funding strategies involves continued uncertainty about future funding, which is aggravated further by the lack of a clear funding policy and therefore a choice set to evaluate the funding needs. The funding strategy also requires decisions on the optimal allocation of funds, where both short-term and long-term benefits must be considered. The low expectations of officials for the program to become self-reliant, and the lack of program champions within the system, may also affect allocations. The absence of fundraising roles within the program structure may also account for the lack of direction in decisions around new funding sources. The final key area – how to achieve more service delivery with less funding – is complex, because it involves multiple interdependent decisions.

**2) Policy development for early infant/early adolescent male circumcision.** Notwithstanding the adjustment of the program focus to older adolescents (aged 15 years and over) alongside EIMC (0–2 months), the goal is still to ensure that the majority of boys are circumcised before they become sexually active. This decision-making phase includes decisions around projections for targets, service delivery, supply chain, decisions to boost demand for EIMC and EAMC among the relevant populations, and decisions around finalization of policy. Decisions on program targets involve providing clarity on data, solving for any ambiguity around indicators, coordination among stakeholders, gaining political consensus, deciding who will take the first step in the hierarchy to initiate the data-gathering process, balancing ministry goals with donor targets, and managing the need for greater accountability across ministry functions and levels. Behavioral dynamics surrounding service delivery projections include disagreements as to which health personnel should provide the service, choosing between efficacy and efficiency, concerns around non-achievement of targets, and managing human resources and infrastructure needs. Decisions for the supply chain involve understanding, managing, and funding inventory in a context where trust and control may be lacking. Boosting demand involves developing new messaging and bringing in other governmental (non-health) stakeholders, while finalizing the policy raises issues of political alignment and will.

**3) Implementation.** This requires policy dissemination and programmatic management, including decisions on client mobilization, supply chain, and service delivery. Decisions in each of these areas involve numerous elements. For policy dissemination, these include the concern that there is more focus on strategy than on actual implementation, while at the same time explaining to stakeholders that the current system is not adequately aligned with user needs. Generating demand for circumcision requires generating new messaging and tailoring it to the required audiences. Supply chain management involves the gradual handover of responsibility for managing inventory funding, and the need to cope with inventory and distribution challenges. Service delivery challenges include questions about the ability of program administrators to manage effectively roles that were previously performed by donor-funded staff, as well as staff capacity and attrition, and changes to incentive structures.

These decision phases consist of a set of iterative decisions, each of which is made under a certain set of conditions or behavioral dynamics which affect the decision by either facilitating it or by becoming a barrier. For example, in the phase of developing an alternative funding strategy we identified four separate decisions, one of which is acknowledging the need for new funding strategies. We identified three conditions or behavioral dynamics affecting this particular decision: a low degree of certainty regarding funding; the evaluation of funding for a policy that has not yet been developed; and a lack of choices, or choices that cannot be compared, increasing the risk due to uncertainty. Each of these three conditions acts as a barrier to actively acknowledging the need for new funding strategies.

The dynamics identified from our interviews appear to be relevant in decision making at one or more levels of the MoH, and may also operate across the MoH and other ministries, implementing partners or donors (as identified by the colored circles attached to each dynamic in Figure 4a– Figure 4c):

1. **National-level:** dynamics involving people, processes, and systems specific to the national MoH
2. **Sub-national-level:** dynamics involving people, processes and systems specific to the provincial- and district-level MoH
3. **Vertical coordination:** dynamics between the national and sub-national levels of the MoH.
4. **Horizontal coordination:** dynamics between the MoH and other stakeholders, including the Ministry of General Education, Ministry of Finance, implementing partners, and donors at each level.

In our example of the decision to acknowledge the need for new funding strategies, the dynamic of low degree of certainty regarding funding was seen to operate at both national and sub-national levels, while the other two dynamics identified (evaluation of funding for a policy that has not been developed yet; and lack of choices, or choices that cannot be compared) were most relevant at the national level.

## Discussion

This study mapped the relevant decision-stakeholders within and supporting Zambia’s health system for transitioning to a sustainable VMMC program, and then used a set of in-depth interviews with a sample of these key decision-makers at all levels of Zambia’s VMMC program to understand the reasons behind delays in the policy-making that is required to transition from a vertical, donor-led VMMC program to one which is government-led and sustainable in the long term.

**Figure 5.**
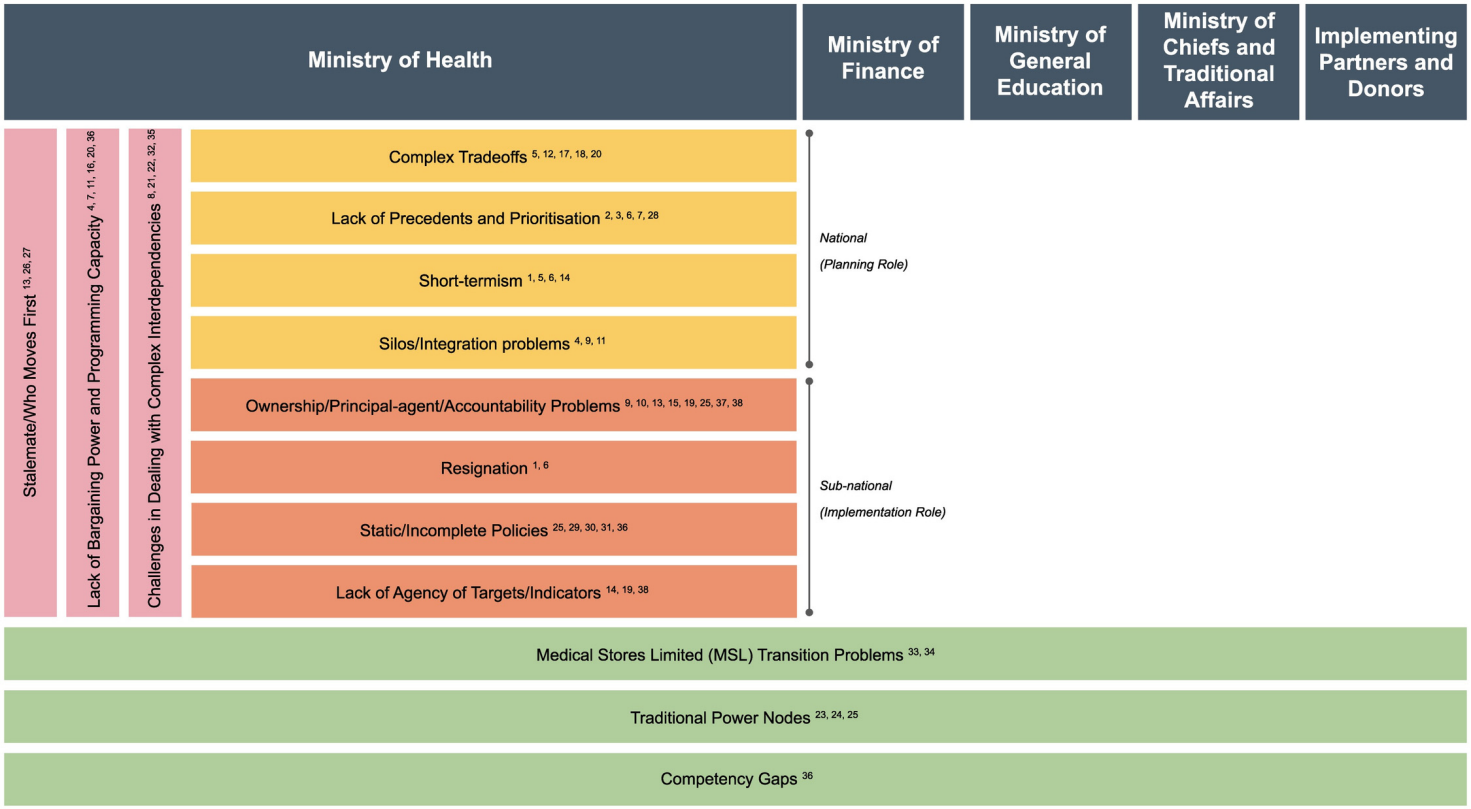
Decision-making barrier framework. * *Yellow: National-level barriers; Orange: Sub-national-level barriers; Pink: Vertical coordination barriers; Green: Horizontal coordination barriers. Numbers in square brackets refer to the conditions/behavioral dynamics in Figure 4a–Figure 4c.*

By analyzing the decision dynamics identified through our interviews, we formulated a framework to understand the barriers that can hinder institutional decision-making and policy development ( Figure 5). The framework sets out four broad categories of barriers that we see as responsible for the observed delay in the transition towards a sustainable VMMC policy.

The **national-level barriers** mostly affect policy development:

  i. **Complex trade-offs:** Policymakers take multiple decisions during policy formulation. These include role allocation, fund allocation, deciding the delivery path, identifying target population, among others. Making trade-offs within these contexts is complex and can lead to delays in the policy development process.
  ii. **Lack of precedents and prioritization:** National level-officials attribute delay in developing a sustainability policy to a lack of reference points for program analysis, cost analysis, transition planning, among others, and hence the need to work from scratch. Prioritization of programs, at both an institutional and systems level, is a particular issue within the MoH, which is responsible for managing a wide variety of health programs. The VMMC program lacks priority within the MoH when pitted against other health programs.
  iii. **Short-termism:** VMMC sustainability depends on long-term strategic initiatives, such as a sustainable “maintenance” phase that includes either or both EIMC and EAMC. At the national level, the potential barrier is the pressure to prioritize short-term gains over long-term gains.
  iv. **Silos and integration problems:** The information required for policy design and development is dispersed within the MoH horizontally and vertically. A systematic perspective is required at the national level but is missing because of the physical and institutional distance between decision-makers within the MoH during policymaking and policy execution.
2. The **sub-national-level barriers** relate to implementation and execution of national policies:
  i. **Ownership/principal-agent/accountability problems:** These revolve around the impact of current incentives for performing VMMCs, the potential removal of incentives, and quality of data. These problems may lessen the willingness to own the program, leading to challenges in implementation, and program direction and management.
  ii. **Resignation:** A successful transition to a sustainable VMMC program is difficult to visualize because of the multiple challenges. The uncertainty around future funding makes it even more difficult to conceive of a successful sustainable program. This results in a feeling of resignation, allowing the inertia of the status quo to win over challenging change.
  iii. **Static/incomplete policies**: The program lacks bottom-up feedback, inclusiveness and representation in the decision-making process. This leads to the development of incomplete or static policies which are not fully relevant or localizable.
  iv. **Lack of agency around targets/indicators:** As program targets are constructed top-down versus bottom-up, healthcare providers and district health officials may interpret targets and indicators differently. This creates a potential barrier to policy sustainability because of the need to increase motivation, and the threat to realizing routinization of VMMC services because of a greater reliance on financial incentives to providers to meet targets.

The coordination barriers involve a more heterogeneous set of stakeholders, processes and systems than the ministry-specific barriers:

3. The **vertical coordination** barriers relate to the inter-level coordination required for policy formulation and execution:
  i. **Stalemate/Who moves first:** There is a lack of clarity among the different stakeholders regarding the order of steps necessary to transition the VMMC program to a sustainable program. Without clarity, individual stakeholders are hesitant to be the first movers toward change.
  ii. **Lack of bargaining power and programming capacity:** Lack of representation from different MoH leadership levels for the program, and lack of decentralized programming capacity at the sub-national levels, hamper buy-in by key institutional MoH decision-makers to advocate for or pursue significant decisions toward sustainability.
  iii. **Challenges in dealing with complex interdependencies:** The challenge of the complicated interdependencies that arise during the development and implementation of a sustainable VMMC program, such as in service delivery where multiple stakeholders are involved, together with the inertia in the system, makes coordinated action toward a sustainable VMMC program difficult.
4. The **horizontal coordination** barriers cut across different ministries within the Government of Zambia relevant for the VMMC policy, as well as donors and implementing partners:
  i. **Competency gaps:** As the VMMC program is expanded into different age cohorts (for example, EIMC), sustainability will depend on the availability of skilled healthcare workers. There must be an emphasis on pre-service training for both general VMMC and EIMC. This re-orientation of the curriculum requires engagement and coordination between the Ministry of General Education and MoH.
  ii. **Traditional power nodes:** This barrier addresses the role played by traditional holders of cultural and community power regarding a sustainable VMMC program. Such power holders include religious leaders, traditional chiefs, etc. and they are linked to the government through the Ministry of Chiefs and Traditional Affairs. Working for sustainability provides both opportunities and challenges in including and collaborating with traditional structures.
  iii. **Medical Stores Limited (MSL) transition problems**: This barrier stems from the need to address supply chain-specific sustainability problems. Moving to a centralized supply chain is seen by many stakeholders as a logical and necessary step toward achieving VMMC sustainability. Because of the nature of the MC supply chain, any changes will involve coordination among stakeholders from different ministries as well as IPs and donors.

Understanding policy development from the perspective of barriers makes it possible to apply a behavioral science-based approach (i.e., behavioral architecture) to address these barriers, at both individual and institutional levels, in order to facilitate successful decision-making, policy development, and program implementation. This is critical because evidence shows that policymakers are not operating with complete information, which hinders their ability to rationally predict policy effects. This leads them to rely on mental models that they have developed from their prior experiences in decision-making. Understanding these drivers of decision-making, and how they play out among the key decision-stakeholders for VMMC sustainability, provides the insights necessary to design a path to sustainability.

Our research did not aim to be exhaustive. Several potential limitations of this study suggest future directions for research. The interviews were conducted with a sample of VMMC program stakeholders from the MoH, other ministries and implementing partners, and while it represented the heterogeneity among the groups, not enough respondents could be interviewed to test and build homogeneity within groups. It also does not completely control for variance within and among geographic provinces. In addition, no qualitative data was collected to create a point of comparison. A larger sample could help identify within-group variance, such as within departments in the MoH, and control for its effects on decision-making. Broadening the sampling frame to include more provinces and districts would also make it possible to control for regional variance, as well as variances among national, provincial, and district levels.

The results of the immersion-phase interviews, and the decision-making barrier framework, served as input for the second phase of our research. To further understand the individual barriers, we developed descriptive and prescriptive hypotheses (see extended data), each of which reflects one or more of the decision dynamics. The descriptive hypotheses aim to explore gaps in understanding around the barriers, while the prescriptive ones help us examine potential interventions based on behavioral principles that can help the transition to a sustainable VMMC program. For example, for the barrier of “Complex trade-offs”, descriptive hypotheses might take the form of questions such as, “What range of options are available to an official developing a health policy?” or “How are decisions made for funding allocations?” Prescriptive hypotheses for the same barrier might include, “Can providing authoritative support help mitigate the problem of complex trade-offs?” and “Can reducing the choice sets available to the officials help them improve their decision heuristics?”

The second phase of research (decision-simulation behavioral research) used exploratory qualitative techniques to refine the descriptive hypotheses and fill our gaps in understanding of systems, processes, and people. We also tested the prescriptive hypotheses using decision stimuli (scenarios) built around specific decision contexts and policy decisions. The coupling between the prescriptive and descriptive hypotheses played an instrumental role in designing decision levers for organizational-level policy for a sustainable VMMC program. This phase of the research is described in a separate article.

Our research adds to the broader understanding of behavioral drivers for policy-making, as well as how to identify those insights. Although the results feed into the specific objectives of the VMMC program, our approach also offers a way of conducting in-depth investigations into organizational-level policy-making. We hope that it will be applicable by HIV programs working in areas other than VMMC, to help them improve decision-making around policy and implementation more broadly.

## Data Availability

Dryad: VMMC Decision Mapping – Phase 1, https://doi.org/10.5061/dryad.z08kprrkz ^40^. This project contains the following underlying data: S1_file.pdf S2_file.xlsx S3_file.xlsx Data are available under the terms of the Creative Commons Zero "No rights reserved" data waiver (CC0 1.0 Public domain dedication).
