# Supplementary material for: Developing a framework for understanding policy decision-making behaviors in the transition of an HIV prevention program towards sustainability: a case study from Zambia’s voluntary medical male circumcision program
Source: Gates Open Res. 2024 Nov 21;8:18. Originally published 2024 Mar 1. [Version 3] doi: 10.12688/gatesopenres.15189.3 (PMC11421490; doi:10.12688/gatesopenres.15189.3)
Supplement: Peer review history

# Reviewer report: Reviewer response for version 3

**DOI:** 10.21956/gatesopenres.17697.r39438

**Published:** 2025-02-25

**Authors:** Joseph Gregory Rosen

**Affiliations:** 1 Brown University, Providence, Rhode Island, USA

This an excellent manuscript that summarizes an application of immersive qualitative research to characterize decision-makers and decision-making pathways to the sustainability of the VMMC program in Zambia. The authors have done an excellent job translating complex narrative data into a well-structured, digestible framework of decision-making in the context of Zambian VMMC programming. I only have a couple of reflections and comments for the authors to consider in a revised manuscript: 

 METHODS

 - In their analysis, can the authors comment on the extent to which their synthesis was team-based? Who was involved specifically in the data synthesis? Was member checking used to help validate the emerging decision-making frameworks or data interpretations?

 DISCUSSION

 - Given the current uncertainties about the future of PEPFAR programming (especially for funding vital prevention services like VMMC), could the authors comment on the utility of such a framework in helping to guide a more rapid transition to program sustainability in this current funding environment?

Is the work clearly and accurately presented and does it cite the current literature?

Yes

If applicable, is the statistical analysis and its interpretation appropriate?

Not applicable

Are all the source data underlying the results available to ensure full reproducibility?

No source data required

Is the study design appropriate and is the work technically sound?

Yes

Are the conclusions drawn adequately supported by the results?

Yes

Are sufficient details of methods and analysis provided to allow replication by others?

Yes

Reviewer Expertise:

HIV epidemiology, HIV care and treatment, implementation science, mixed-methods research

I confirm that I have read this submission and believe that I have an appropriate level of expertise to confirm that it is of an acceptable scientific standard.

# Reviewer report: Reviewer response for version 3

**DOI:** 10.21956/gatesopenres.17697.r38501

**Published:** 2024-11-26

**Authors:** Robin Schaefer

**Affiliations:** 1 Forum for Collaborative Research, University of California Berkeley, Berkeley, California, USA

I thank the authors for addressing my previously raised comments. I have no further feedback and I think the article represents a good contribution to the literature.

Is the work clearly and accurately presented and does it cite the current literature?

Yes

If applicable, is the statistical analysis and its interpretation appropriate?

I cannot comment. A qualified statistician is required.

Are all the source data underlying the results available to ensure full reproducibility?

No source data required

Is the study design appropriate and is the work technically sound?

Yes

Are the conclusions drawn adequately supported by the results?

Yes

Are sufficient details of methods and analysis provided to allow replication by others?

Yes

Reviewer Expertise:

NA

I confirm that I have read this submission and believe that I have an appropriate level of expertise to confirm that it is of an acceptable scientific standard.

# Reviewer report: Reviewer response for version 2

**DOI:** 10.21956/gatesopenres.17435.r38091

**Published:** 2024-09-17

**Authors:** Robin Schaefer

**Affiliations:** 1 Forum for Collaborative Research, University of California Berkeley, Berkeley, California, USA

I thank the editors for inviting me to review this interesting article on a framework to understand decisions that can support or hinder the transition of Zambia’s VMMC program from a donor-funded to a government-owned program. The authors conducted a range of qualitative interviews to develop a comprehensive framework. The article is well-written, with conclusions well supported by the collected data. The approach and results do not just support this specific program but are transferable to other programs and settings, so the article is likely of interest to a broad range of readers. Below are some suggestions to further strengthen the article.

 Introduction

 - Second paragraph: To adequately reflect the results of the trials on VMMC, I suggest removing “unprotected”. The high-level 60% risk reduction result was the result of an intent-to-treat analysis (and there was condom use in the intervention and control groups).

 - Third paragraph: The authors refer to VMMC as a “cost-effective intervention”. Please provide a reference to substantiate the claim of cost-effectiveness.

 - Final paragraph: The authors may want to mention a few details on their research, e.g. what methods were used and what were the goals. Some of this is mentioned in the next section.

 Program sustainability and decision-making

 - Final paragraph: I suggest integrating  background on VMMC in Zambia and the research into the introduction.

 Methods

 - A separate article is mentioned on phase two methods and results. Please include a reference.

 - What methods were used to map the organizational structures as relevant for the VMMC program? E.g., what data sources were used?

 - It would be helpful if the semi-structured interview guide could be included as an appendix.

 Discussion

 - The approach the authors applied in their research and their resulting framework could be usefully applied to other programs or areas. This is mentioned in the introduction and I suggest to reflect on this further in the discussion.

Is the work clearly and accurately presented and does it cite the current literature?

Yes

If applicable, is the statistical analysis and its interpretation appropriate?

I cannot comment. A qualified statistician is required.

Are all the source data underlying the results available to ensure full reproducibility?

No source data required

Is the study design appropriate and is the work technically sound?

Yes

Are the conclusions drawn adequately supported by the results?

Yes

Are sufficient details of methods and analysis provided to allow replication by others?

Yes

Reviewer Expertise:

NA

I confirm that I have read this submission and believe that I have an appropriate level of expertise to confirm that it is of an acceptable scientific standard, however I have significant reservations, as outlined above.

# Response

**Published:** 2024-11-20

**Authors:** Nishan Gantayat

**Affiliations:** Behavioral Science, Not Applicable, Mumbai, Maharashtra, India

**Reviewer: **Introduction:

 Second paragraph: 

 To adequately reflect the results of the trials on VMMC, I suggest removing “unprotected”. The high-level 60% risk reduction result was the result of an intent-to-treat analysis (and there was condom use in the intervention and control groups). 

*Author's response - Noted and edits made.*

**Reviewer: **Third paragraph: 

 The authors refer to VMMC as a “cost-effective intervention”. Please provide a reference to substantiate the claim of cost-effectiveness.

*Author's response - Noted and edits made.*

**Reviewer: **Final paragraph: 

 The authors may want to mention a few details on their research, e.g. what methods were used and what were the goals. Some of this is mentioned in the next section.Program sustainability and decision-making

*Author's response - Noted and edits made.*

**Reviewer: **Final paragraph: I suggest integrating background on VMMC in Zambia and the research into the introduction.

*Author's response - Noted. We did try to integrate the background in the introduction but it was causing breaks in the flow and needed major structural revisions. Since the details are all out there, we have chosen not to disturb the structure.*

**Reviewer: **Methods: A separate article is mentioned on phase two methods and results. Please include a reference.

*Author's response - Noted. The paper manuscript is being worked on and is a work in progress.*

**Reviewer:**What methods were used to map the organisational structures as relevant for the VMMC program? E.g., what data sources were used? -

*Author's response - Noted and edits made. Have provided additional information in the text.*

**Reviewer : **It would be helpful if the semi-structured interview guide could be included as an appendix.

*Author's response - Noted. The guide has been included as part of the open access supplementary materials during submission as per the guidelines of the journal.*

**Reviewer: **Discussion: The approach the authors applied in their research and their resulting framework could be usefully applied to other programs or areas. This is mentioned in the introduction and I suggest to reflect on this further in the discussion.

*Author's response - Noted. Edits made and text added in the last paragraph.*

# Reviewer report: Reviewer response for version 2

**DOI:** 10.21956/gatesopenres.17435.r37149

**Published:** 2024-07-19

**Authors:** Edinah Mudimu

**Affiliations:** 1 University of South Africa, Pretoria, Gauteng, South Africa

The authors have addressed all my comments. I have no further comments to make.

Is the work clearly and accurately presented and does it cite the current literature?

Yes

If applicable, is the statistical analysis and its interpretation appropriate?

Yes

Are all the source data underlying the results available to ensure full reproducibility?

Yes

Is the study design appropriate and is the work technically sound?

Yes

Are the conclusions drawn adequately supported by the results?

Yes

Are sufficient details of methods and analysis provided to allow replication by others?

Yes

Reviewer Expertise:

Mathematical modelling of infectious diseases

I confirm that I have read this submission and believe that I have an appropriate level of expertise to confirm that it is of an acceptable scientific standard.

# Reviewer report: Reviewer response for version 1

**DOI:** 10.21956/gatesopenres.16533.r36106

**Published:** 2024-07-19

**Authors:** Elijah Odoyo-June

**Affiliations:** 1 CDC DGHT, Center for disease control and prevention (CDC), Kenya Place: Kisumu, Kisumu, Kisumu, Kenya; 2 Center for Global Health, Kisumu, Kenya

**Summary**

 The authors interviewed VMMC stakeholders in Zambia to understand how the behaviors and non-conscious thought processes of decision makers in the Ministry of Health could hamper VMMC transition from a donor driven to a country-led sustainable program. They identified three decision-making phases in VMMC program transition, namely, 1) developing an alternative funding strategy, 2) age prioritization for VMMC services and 3) identifying efficient implementation models. For each phase they identified potential barriers to effective decision-making.

**General**

 In the introduction, key elements of program sustainability are identified from World bank, USAID and PEPFAR perspectives and examples of transition to sustainable HIV programming from different regions of the world are cited. The manuscript is clearly written and addresses an important topic. It could however benefit from some edits. Based on the core elements of sustainability presented in the introduction, the possibility of identifying the key decision-making stages and potential barriers through intuition cannot be excluded. The meticulous processes taken to achieve the objectives of the project may be simplified in future for practical application in program transition efforts for long term sustainability.

**Introduction**

 Paragraph 1

 The second sentence in paragraph 1 under introduction should be edited to improve its clarity.

 It is stated that the resource pool for low- and middle-income counties was $ 21.7bn in 2020. It also stated in the same paragraph that donor funding to these countries declined to $ 8.2bn in 2020. The difference between $21.7 and $8.2bn in 2020 in 2020 should be made clear.

 Paragraph 3

 Consider editing the first sentence in the 3 ^rd^ paragraph under introduction to show that donor funding is has not just plateaued but is declining.

 Also consider editing the second sentence in the 3 ^rd^ paragraph to show that what is needed for VMMC sustainability goes beyond routing donor funds through government finance mechanisms and service integration to include the use of domestic resources to support the program.

**Methods**

 Out of the 21 purposively selected participants interviewed, 17 were individuals with key decision-making roles for the transition of the VMMC program in Ministry of health. For each level (National, Provincial and District), a brief description of how the decision-making roles were weighted for inclusion in the study may be helpful especially to guide reproducibility of the case study in other settings.

Is the work clearly and accurately presented and does it cite the current literature?

Yes

If applicable, is the statistical analysis and its interpretation appropriate?

Not applicable

Are all the source data underlying the results available to ensure full reproducibility?

Yes

Is the study design appropriate and is the work technically sound?

Yes

Are the conclusions drawn adequately supported by the results?

Partly

Are sufficient details of methods and analysis provided to allow replication by others?

Partly

Reviewer Expertise:

Public health epidemiology

I confirm that I have read this submission and believe that I have an appropriate level of expertise to confirm that it is of an acceptable scientific standard, however I have significant reservations, as outlined above.

## References

[1] : Developing a framework for understanding policy decision-making behaviors in the transition of a HIV prevention program towards sustainability: a case study from Zambia’s voluntary medical male circumcision program. *Gates Open Research* .2024;8: 10.12688/gatesopenres.15189.1 10.12688/gatesopenres.15189.1 PMC1142149039319309

# Response

**Published:** 2024-11-20

**Authors:** Nishan Gantayat

**Affiliations:** Behavioral Science, Not Applicable, Mumbai, Maharashtra, India

Thank you for your valuable feedback. Please find below my response. The updated manuscript has been uploaded. 

**Introduction**

 Paragraph 1

 The second sentence in paragraph 1 under introduction should be edited to improve its clarity.

 It is stated that the resource pool for low- and middle-income counties was $ 21.7bn in 2020. It also stated in the same paragraph that donor funding to these countries declined to $ 8.2bn in 2020. The difference between $21.7 and $8.2bn in 2020 in 2020 should be made clear.

*Author's Reponse - Noted and edits made.*

 Paragraph 3

 Consider editing the first sentence in the 3rd paragraph under introduction to show that donor funding is has not just plateaued but is declining.

*Author's Reponse - Noted and edits made.*

 Also consider editing the second sentence in the 3rd paragraph to show that what is needed for VMMC sustainability goes beyond routing donor funds through government finance mechanisms and service integration to include the use of domestic resources to support the program. 

*Author's Reponse - Noted and edits made.*

**Methods**

 Out of the 21 purposively selected participants interviewed, 17 were individuals with key decision-making roles for the transition of the VMMC program in Ministry of health. For each level (National, Provincial and District), a brief description of how the decision-making roles were weighted for inclusion in the study may be helpful especially to guide reproducibility of the case study in other settings.

*Author's Reponse - Noted and edits made.*

# Reviewer report: Reviewer response for version 1

**DOI:** 10.21956/gatesopenres.16533.r36109

**Published:** 2024-04-05

**Authors:** Edinah Mudimu

**Affiliations:** 1 University of South Africa, Pretoria, Gauteng, South Africa

The manuscript titled “Developing a framework for understanding policy decision-making behaviors in the transition of a HIV prevention program towards sustainability: a case study from Zambia’s voluntary medical male circumcision program” addresses an important issue especially in this era where HIV funding for low- and middle-income countries is declining. Despite the decline in funding, HIV remains a pressing health issue that necessitates continued funding to achieve the goal of ending HIV as a public health threat by 2030. The authors did a commendable job of conducting interviews with key stakeholders at various levels (national, provincial, and district) of the Ministry of health and with stakeholders (donors and partners} funding Zambia’s voluntary medical male circumcision (VMMC) program to gather their perspectives on achieving a sustainable VMMC program with limited or without donor funding. While I am not an expert in the methodology employed to analyse collected data (pattern identification and theme matching), the analysis is clearly explained, and all data is shared with the readers demonstrating transparency in the analysis process. In addition to my following insights, I suggest seeking input from a specialist in organizational development and the formulation of transition plans for policymaking. Specific comments to the authors are as follows:

1. Title: minor edit – replace “…a HIV prevention…” with “…an HIV prevention…”
2. Keywords: capitalise “z” for the word “Zambia”

Introduction

1. Delete “and” in the following “…supply-side focused, and donor-funded…’
2. I suggest that the sentence regarding Rwanda's capacity building follows the subsequent sentence concerning India's prevention initiative. This adjustment will enhance the readability of the text since the authors mention South Africa and India, and not Rwanda, in the two preceding sentences before the one in question.
3. Maybe necessary to add and in the following “sero-discordant couples, and in Zambia using a “train-the-trainer” model”

Methods

1. Authors mention that the methods and results for Phase Two are reported in a separate article (also mentioned in the last sentence of the second last paragraph in the Discussion section). A reference is necessary if the Phase Two work has been published. If not yet published it will help if the authors indicate that it is work in progress.
2. Authors used purposive sampling to select individuals with key roles for the transition of the VMMC program. Using purposive sampling makes it possible for researchers to tailor their sampling approach to the unique characteristics of the research study which applies well in this research study however authors should keep in mind that it may be challenging to generalize findings from this study beyond the specific context from which the sample was drawn.

Supplementary file S3

1. There is information duplication row 4 and row 25 column I. Consider deleting one of the two.

General comments: Overall the manuscript is well written with minor editorial errors which can be fixed by reading through the article thoroughly.

Is the work clearly and accurately presented and does it cite the current literature?

Yes

If applicable, is the statistical analysis and its interpretation appropriate?

Yes

Are all the source data underlying the results available to ensure full reproducibility?

Yes

Is the study design appropriate and is the work technically sound?

Yes

Are the conclusions drawn adequately supported by the results?

Yes

Are sufficient details of methods and analysis provided to allow replication by others?

Yes

Reviewer Expertise:

Mathematical modelling of infectious diseases

I confirm that I have read this submission and believe that I have an appropriate level of expertise to confirm that it is of an acceptable scientific standard, however I have significant reservations, as outlined above.

# Response

**Published:** 2024-06-06

**Authors:** Nishan Gantayat

**Affiliations:** Behavioral Science, Not Applicable, Mumbai, Maharashtra, India

Thank you for the peer review. We have been able to incorporate the changes and have submitted a new version with all the changes incorporated, barring the suggestion of capitalizing "Z" in Zambia in the keyword section, which we were not able to do while submitting the new version. We have asked the editors to assist us on that.
